# Thalamic iron in multiple sclerosis: Waning support for the early-rise late-decline hypothesis

**DOI:** 10.1016/j.nicl.2025.103771

**Published:** 2025-03-26

**Authors:** Fahad Salman, Niels Bergsland, Michael G. Dwyer, Jack A Reeves, Abhisri Ramesh, Dejan Jakimovski, Bianca Weinstock-Guttman, Robert Zivadinov, Ferdinand Schweser

**Affiliations:** a Buffalo Neuroimaging Analysis Center, Department of Neurology at the Jacobs School of Medicine and Biomedical Sciences, University at Buffalo, The State University of New York, Buffalo, NY, United States; b Department of Biomedical Engineering, University at Buffalo, The State University of New York, Buffalo, NY, United States; c Center for Biomedical Imaging, Clinical and Translational Science Institute, University at Buffalo, The State University of New York, Buffalo, NY, United States; d Wynn Hospital, Mohawk Valley Health System, Utica, NY, United States; e Jacobs Neurological Institute, Buffalo, NY, United States

**Keywords:** Multiple sclerosis, quantitative susceptibility mapping (QSM), Thalamus, Iron metabolism, Reproducibility, Deep gray matter

## Abstract

**Background:**

Studies of thalamic iron levels in multiple sclerosis (MS) have yielded variable findings, potentially due to differences in study cohorts. For example, studies in relatively young cohorts (average ages below 40 years) have reported elevated susceptibility in people with MS (pwMS), whereas studies in older cohorts (above 40 years) found decreased susceptibility.

**Objective:**

To test the “early-rise late-decline” hypothesis, which posits that age differences in study cohorts are responsible for conflicting findings regarding thalamic susceptibility in MS.

**Methods:**

We chose to replicate one of the previous studies that showed evidence of elevated thalamic iron concentrations in younger pwMS (Rudko et al., 2014). We also replicated a study involving older pwMS (Pudlac et al., 2020) to serve as a control. We assessed thalamic susceptibility using the QSM processing and analysis methodology outlined by Rudko et al.

**Results:**

Although cohort characteristics, QSM processing, and analytical methods were closely matched, we found significantly lower thalamic susceptibility in the younger pwMS compared to controls (−1.1 ± 7.8 vs. 5.4 ± 6.1 ppb; effect sizes: −0.35 to −0.91). Study outcomes were robust across a wide range of regularization parameters, with effect size differences influenced by background field removal regularization. A similar pattern was observed in the older cohort, where thalamic susceptibility was again lower in pwMS compared to controls (4.0 ± 9.5 vs. 9.6 ± 10.7 ppb; effect size: −0.55).

**Conclusions:**

Our findings contradict the “early rise” hypothesis of thalamic iron levels in pwMS. The consistency of our results across multiple analyses suggests that QSM processing artifacts are unlikely to explain previous reports of increased thalamic iron. Instead, these variations may stem from demographic or clinical differences, such as geographical factors and treatment regimens.

## Highlights

• Tested early-rise late-decline thalamic susceptibility hypothesis in multiple sclerosis by reproducing prior study.
• Our study suggests decreased susceptibility in the thalamus can occur early in the disease.
• Choice of the BFR regularization parameter had the strongest effect on study outcomes.

## 1 Introduction

Multiple sclerosis (MS) is a neurodegenerative disorder characterized by demyelination and disturbed brain iron levels, particularly in the deep gray matter (DGM) (Haider et al., 2014, Filippi et al., 2019, Hagemeier et al., 2012, Vercellino et al., 2009, Zecca et al., 2004, Stankiewicz et al., 2014). This disturbance may play a critical role in MS pathophysiology (Burgetova et al., 2017, Hagemeier et al., 2018, Langkammer et al., 2013, Schweser et al., 2018, Zivadinov et al., 2018, Khalil et al., 2015, Schweser et al., 2021, Rudko et al., 2014, Pudlac et al., 2020); as iron is an essential co-factor in many biochemical processes of the brain, including myelin maintenance (Rouault, 2013).

Quantitative susceptibility mapping (QSM) has emerged as one of the most sensitive clinical methods for assessing brain iron alterations (Langkammer et al., 2012, Voon et al., 2024). Two recent systematic reviews (Voon et al., 2024, De Lury et al., 2023) demonstrated that QSM studies consistently reported either no changes or *elevated* susceptibility throughout most of the DGM in people with MS (pwMS) compared to healthy controls, suggesting elevated iron concentrations. Other iron-sensitive MRI metrics, such as the effective transverse relaxation rate (R_2_*), showed similar patterns (De Lury et al., 2023). The reviews, however, found greater variability in thalamic findings. For example, while some studies observed no significant differences (Langkammer et al., 2013, Al-Radaideh et al., 2013, Burgetova et al., 2017, Elkady et al., 2017, Elkady et al., 2019, Fujiwara et al., 2017, Cho et al., 2022, Elkady et al., 2018) or reported thalamic susceptibility alterations suggestive of *elevated* iron (some R_2_*-based studies (Walsh et al., 2014, Quinn et al., 2014, Lebel et al., 2012) and two studies (Rudko et al., 2014, Cobzas et al., 2015) using both QSM and R_2_*), most studies supported *reduced* (not increased) (Burgetova et al., 2017, Hagemeier et al., 2018, Schweser et al., 2018, Zivadinov et al., 2018, Schweser et al., 2021, Pudlac et al., 2020, Bergsland et al., 2018, Hagemeier et al., 2018, Pontillo et al., 2019) iron concentrations in pwMS (Voon et al., 2024, De Lury et al., 2023). The evidence provided by the two studies with both QSM and R_2_* represented particularly robust support of elevated thalamic iron in pwMS. Although R_2_* is derived from the same pulse sequence as QSM, it quantifies a complementary physical effect with less complex signal processing, making it a semi-independent metric. Furthermore, while both R_2_* and susceptibility have a similar dependence on iron—more iron increases both metrics (Langkammer et al., 2012, Langkammer et al., 2010)—the bias from concomitant myelin rarefaction is opposite, with rarefaction decreasing R_2_* and increasing susceptibility (Langkammer et al., 2012).

In 2018, Schweser et al. (Schweser et al., 2018) observed that the cohorts in studies suggesting elevated thalamic iron in pwMS were on average younger (e.g., 37.3 ± 6.1 (Rudko et al., 2014) and 35.6 [±std not reported] (Cobzas et al., 2015) years) than in studies that reported reduced thalamic susceptibility (e.g., 43 ± 10.1 (Hagemeier et al., 2018) to 57 ± 12.1 (Bergsland et al., 2018) years average). This observation led to the formulation of a phenomenological hypothesis about the trajectory of thalamic iron in pwMS known as the “early-rise late-decline” hypothesis (Schweser et al., 2018). Drawing from the cumulative findings of imaging studies, the authors posited that *peak iron* concentrations in the thalamus occur early in the life of pwMS and hence may be a potential early imaging biomarker for MS (Schweser et al., 2018). While no direct histopathological evidence was available, the authors discussed several potential mechanistic pathways leading to the early-rise and late-decline phases (Schweser et al., 2018). Given the influence of these observations, the lack of supporting studies in the past decade, and the fact that all studies supporting increased thalamic iron originated from Canada, we deemed it essential to reproduce previous findings of “early-rise” in pwMS.

Building on earlier hypotheses and findings (Schweser et al., 2018); we hypothesized that the previously observed increase in thalamic susceptibility in younger pwMS (Rudko et al., 2014, Cobzas et al., 2015) could be reproduced in an independent study, provided that cohort characteristics, QSM reconstruction methodology, and analysis procedures matched the original studies. This hypothesis is grounded in the general scientific principle that research outcomes should be reproducible under (nearly) identical experimental conditions. Here, we focused on reproducing Rudko et al.’s study, which had a larger effect size (*d* = 0.86) than Cobzas et al.’s (*d =* 0.58) (Voon et al., 2024). As a control, we also aimed to reproduce a QSM study that found decreased thalamic susceptibility in an older cohort. We chose the 2020 study by Pudlac and colleagues (Pudlac et al., 2020) because it is a relatively recent study with one of the oldest average cohort ages (47.0 ± 9.0 years) and was published by a group other than ours. We enrolled participants for cohorts that closely matched the clinical and demographic characteristics of the original studies (Rudko et al., 2014, Pudlac et al., 2020). To enable direct comparison, we applied Rudko et al.’s QSM processing pipeline to the cohort matching Pudlac et al.’s study. The early-rise-late-decline hypothesis would be supported if we observed significantly elevated thalamic susceptibility in younger pwMS and significantly reduced susceptibility in older pwMS; otherwise it would be refuted.

## 2 Methods

### 2.1 Study participants

We selected two cohorts from a large institutional database of scans collected in previous IRB-approved studies. Written informed consent was obtained from all participants in compliance with Health Insurance Portability and Accountability Act (HIPAA) regulations. The cohorts were designed to align with the group-level clinical and demographic characteristics reported in the original studies by Rudko et al. and Pudlac et al., specifically average and standard deviation of age, sex ratio, and for pwMS, phenotype, average and standard deviation of disease duration, and average and range (standard deviation for Pudlac et al. [*older cohort* in this study]) of Expanded Disability Status Scale (EDSS). Healthy controls underwent a normal neurological examination and had no history of neurological or chronic psychiatric disorders at the time of scanning.

For the Rudko et al. matched cohort, referred to as the *younger cohort* in this study, we tripled the sample size to compensate for the lower signal-to-noise ratio in our QSM data due to the reduced field strength (3T vs. 7T) compared to the original study. We kept the ratios of people with clinically isolated syndrome (CIS) to people with relapsing MS (RMS) similar to the original study (4:21), as well as the ratio of controls to pwMS (15:25). This led to an enrollment of 13 people with CIS, 70 people with RMS, and 44 controls.

Additionally, to assess whether Rudko et al.’s findings were influenced by the inclusion of people with CIS, who may not progress to MS and could bias the study toward non-MS effects, we created a third cohort comprising only individuals with RMS. This was achieved by replacing all CIS subjects (*N* = 13) and a few people with RMS (4) to maintain clinical and demographic characteristics of the group. Referred to as the *younger cohort without CIS*, it included 83 people with RMS and 44 controls, with the majority of people with RMS (66) and all controls overlapping with the originally matched cohort.

### 2.2 Imaging

All scans were acquired using the same 3T MRI scanner (Signa Excite HD 12.0; General Electric, Milwaukee, WI, USA) over a period with no hardware or software upgrades. The scanner used an eight-channel head-and-neck coil. In addition to 3D T1-weighted (T1w) imaging using previously described parameters (Hagemeier et al., 2018); the raw k-space data for QSM were acquired using an unaccelerated 3D single-echo spoiled gradient recalled echo sequence with first-order flow compensation in read and slice directions, a matrix of 512 × 192 × 64 and a nominal resolution of 0.5 × 1 × 2 mm^3^ (FOV = 256 × 192 × 128 mm^3^), flip angle = 12°, TE/TR = 22 ms/40 ms, bandwidth = 13.89 kHz, acquisition time = 8:46 min:seconds. Table 1 contrasts the acquisition parameters of the present study with those of the two original studies.

**Table 1:** Comparison of gradient echo (GRE) sequence acquisition parameters between the present and original studies (Rudko et al. and Pudlac et al.; NR = not reported).

	Present study	Rudko et al.	Pudlac et al.
Field strength (Tesla [T])	3	7	1.5
Repetition time (TR; ms)	40	40	48.1
Echo time (TE; ms)	22	3.77:Δ4.30:26.22	33.2
Flip angle (°)	12	13	NR
Voxel size (mm^3^)	0.5 × 1 × 2	0.5 × 0.5 × 1.25	0.8 × 0.8 × 2
Matrix dimensions	512 × 192 × 64	380 × 340 × 102	NR
Field of View (FOV; mm^3^)	256 × 192 × 128	190 × 170 × 128	NR
Imaging Time (min)	8.76	15.92	6.50

### 2.3 QSM processing

Younger cohorts

The raw k-space data for QSM were processed into field maps following previously described methods (Hagemeier et al., 2018). We sought to precisely replicate the QSM reconstruction and analysis procedures used by Rudko et al. Specifically, we utilized Sophisticated Harmonic Artifact Reduction for Phase data (SHARP (Schweser et al., 2011) with a 7 mm radius for background field removal (BFR), Morphology Enabled Dipole Inversion (MEDI (Liu et al., 2012) for dipole inversion, FSL‐FIRST from the FMRIB Software Library (Patenaude et al., 2011) for DGM segmentation, and frontal deep white matter (FDWM) as a reference region for susceptibility values.

*Brain mask*

As Rudko et al. did not provide a detailed QSM-based masking procedure, we followed all masking procedures in compliance with the recent 2024 QSM Consensus Recommendations (Consensus Organization Committee Bilgic, et al., 2024); as illustrated in the study-specific schematic provided in Supplementary [Supp. Fig. 1].

The initial brain mask (*mask 1*; Supp. Fig. 1) was generated by applying a whole-brain segmentation tool – FSL-brain extraction tool (BET (Jenkinson et al., 2012) to the magnitude images. This mask removed air, skull, and other tissues while preserving cortical areas. Following this, a mask of reliable phase values (*mask 2*) was generated by thresholding the two-pixel finite difference of the unwrapped phase at an empirically determined value of 2.6 rad. Subsequently, *masks 1* and *2* were logically combined, and holes were filled by first dilating the mask and then performing erosion from the outer boundary to obtain *mask 3*. This resultant mask was used for the BFR step (Supp. Fig. 1).

*Background field removal*

Rudko et al.’s study did not explicitly define a regularization parameter for SHARP. To account for the potential range of the implicitly defined regularization values used in the original publication, we applied SHARP with a broad spectrum of regularization (λ_B_) parameters and repeated all subsequent processing steps for all resulting phase images. We uniformly distributed 14 λ_B_-values in log-scale, ranging from 0.0031 to 0.0430. For improved comparability with other studies, we used the resolution-independent parameter scaling definition recommended by Özbay et al. (Özbay et al., 2017).

The BFR algorithm created an eroded subject mask (*mask 4*). We logically combined *masks 2* and *4* to create a mask for the dipole inversion step, *mask 5* (Supp. Fig. 1).

*Dipole inversion*

The regularization parameter used in the original study for MEDI was not documented. While the authors followed an L-curve approach (de Rochefort et al., 2010) (personal communication), the final regularization parameter for the optimization was not saved. As with SHARP, to address this, we applied MEDI with a wide range of regularization parameters (λ_D_; 14 values between 1 and 100,000) to all phase images obtained with the 14 λ_B_ parameters. We then repeated all subsequent analytical steps (see 2.4, 2.5 below) for all resulting susceptibility maps. In summary, we compared combinations of the 14 λ_B_ and 14 λ_D_ for both younger cohorts, resulting in a total of 28,224 susceptibility maps across 144 subjects (127 subjects for the younger cohort and 17 in the RMS-only cohort).

Throughout the remainder of this work, we refer to each algorithmic configuration (specific set of λ_B_ and λ_D_ parameters) as a QSM “pipeline” (196 pipelines).

Older cohort

We processed the older cohort using two pipelines: (i) the pipeline with the *highest* absolute thalamus effect size in the younger cohort, and (ii) the pipeline that had the *smallest* absolute thalamus group difference in the younger cohort. The second pipeline was included to avoid a selection bias toward a study outcome of thalamic group difference. Both pipelines were expected to output a total of 120 reconstructed susceptibility maps across 60 subjects (20 controls and 40 pwMS).

### 2.4 Region-of-interest analysis

*Regions of interest*

Our primary analysis focused on the thalamus. A secondary analysis examined the globus pallidus (GP), caudate, and putamen to evaluate whether findings in these regions were consistent between studies. The region-of-interest (ROI) selection for the secondary analysis was in accordance with Rudko et al.’s ROIs. It should be noted that, while Rudko et al. also investigated thalamic subregions using a voxel-wise analysis, we focused our efforts only on findings relating to the whole thalamus.

*FSL FIRST segmentation*

FIRST was applied to each subject’s native T1w image without lesion filling as it has been shown that lesions have a negligible impact on the FIRST segmentation outcomes (González-Villà et al., 2017). Subsequently, the subject-specific DGM ROIs were extracted from the FIRST labels and transformed onto the subjects’ native QSM space. To this end, we used FSL FLIRT (Jenkinson et al., 2012) with 6 degrees-of-freedom to rigidly align magnitude GRE images to their respective T1w images, leveraging the T1w images' superior cortical contrast, and applied the inverse of the resulting transformation matrix to ROIs with nearest neighbour interpolation. The ROIs were then binarized using fslmaths. Bi-lateral regional mean susceptibility values were calculated using fslstats (Jenkinson et al., 2012).

*Referencing*

We used the diffeomorphic Greedy-SyN transformation model in Advanced Normalization Tools (Avants et al., 2009) (version 2.1; https://stnava.github.io/ANTs) to generate a study-specific bi-parametric (QSM-T1w; Supp. Fig. 2) template as previously described (Hanspach et al., 2017). A trained analyst (F.Sa.) delineated the FDWM region on the QSM contrast of the bi-parametric template; this region was then propagated to the subjects' native QSM spaces using subject-specific warp field computations. Following this, all regional mean susceptibility values were referenced to the FDWM.

### 2.5 QSM analysis

*Statistical analysis*

For each pipeline, bi-lateral referenced ROI measures were averaged across hemispheres and corrected for age-dependent changes by subtracting the effect of age from the QSM findings, following the method described by Rudko et al. Subsequently, all age-corrected regional findings underwent independent t-tests to assess group differences between pwMS and controls.

*Effect sizes*

We calculated Cohen’s *d* effect sizes for quantitative assessment of the impact of different parameter choices on study outcomes. The effect size (*d*) quantifies the strength of an observation in a cohort-based study.

*Visualization*

Effect sizes for all pipelines were visualized as heat maps using the Seaborn library in Python 3.0 (Waskom, 2021).

### 2.6 Scientific rigor

*QSM processing*

To ensure accurate implementation, all QSM processing used code provided by the original developers. To ensure high scientific rigor, we performed all processing fully automated and reproducible using containerized computing on a high-performance cluster allocating 4 cores (Intel Xeon Gold 6330) and variable memory, depending on the algorithm (4.5 and 24 GB for SHARP and MEDI, respectively), to each job. We used an in-house developed job scheduler for multi-step SLURM (Simple Linux Utility for Resource Management) pipelines with dependencies (pi4s; https://gitlab.com/R01NS114227/pi4s). All QSM related processes were executed in a custom Singularity container generated with Neurodocker (https://www.repronim.org/neurodocker/), ensuring that the computational environment was identical for all methods. The container included MATLAB (version 2018b; The MathWorks, Natick, MA), FreeSurfer (v6.0.0), FSL (v5.0.8), and ANTs (v2.0.0).

*Quality Control*

Subject-specific native space QSM montages were generated using MATLAB (version 2018b; The MathWorks, Natick, MA), with DGM and FDWM label overlays to verify ROI placement. This quality control step ensured accurate segmentation mask placement over the intended regions on susceptibility maps.

## 3 Results

### 3.1 Participants’ demographics and clinical characteristics

The younger cohort had an mean age (±standard deviation) of 37.4 ± 4.5 years and an EDSS of 1.7 (range: 0–6.0). For the younger cohort without CIS, we replaced all individuals with CIS (*N* = 13) and four additional RMS subjects, resulting in a mean age of 37.0 ± 4.5 years and an EDSS of 1.7 (range 0–3). The age- and sex-matched healthy control group (*N* = 44) had an average age of 36.9 ± 4.5 years. The older cohort had an average age of 46.0 ± 9.3 years, EDSS of 3.2 ± 1.8, and a disease duration of 19.3 ± 4.2 years. All subjects were scanned between 2008 and 2019. Cohort characteristics are summarized and compared with the original studies in Table 2, Table 3.

**Table 2:** Demographic and clinical characteristics of the Rudko et al.’s original cohort compared to the present study’s younger cohorts.

			Present study			
	Rudko et al.		CIS & RMS		only RMS	
Variables	Controls	pwMS	Controls	pwMS	Controls	pwMS
Subjects	15	25	44	83	44	83
M:F Sex Ratio	1:4	1:2.5	1:3	1:2.6	1:3	1:2.6
Age^†^	36.4 ± 6.4	37.3 ± 6.1	36.9 ± 4.5	37.4 ± 4.5	36.9 ± 4.5	37.0 ± 4.5
Disease Duration^†^	−	NR	−	7.4 ± 6.6	−	8.1 ± 6.9
CIS:RMS ratio	−	4/21	−	13/70	−	0/83
EDSS^‡^	−	1.7 (0–6.0)	−	1.7 (0–6.0)	−	1.7 (0–3.0)

† Data are means ± standard deviations.

‡ Numbers in parentheses represent range of scores for all pwMS.

NR = not reported.

**Table 3:** Demographic and clinical characteristics of Pudlac et al.’s original cohort compared to the present study’s older cohort. All pwMS (*N =* 40) in both studies were diagnosed with RMS.

	Pudlac et al.		Present study	
Variables	Controls	pwMS	Controls	pwMS
Subjects	20	40	20	40
M:F Sex Ratio	1:4	1:4	1:4	1:4
Age^†^	50.0 ± 8.0	47.0 ± 9.0	49.3 ± 8.9	46.0 ± 9.3
Disease Duration^†^	−	14.1 ± 8.2	−	19.3 ± 4.2
EDSS^†^	−	3.1 ± 1.6	−	3.2 ± 1.8

† Data are means ± standard deviations.

### 3.2 Visual inspection of regularization impact and ROIs

**Fig. 1:**
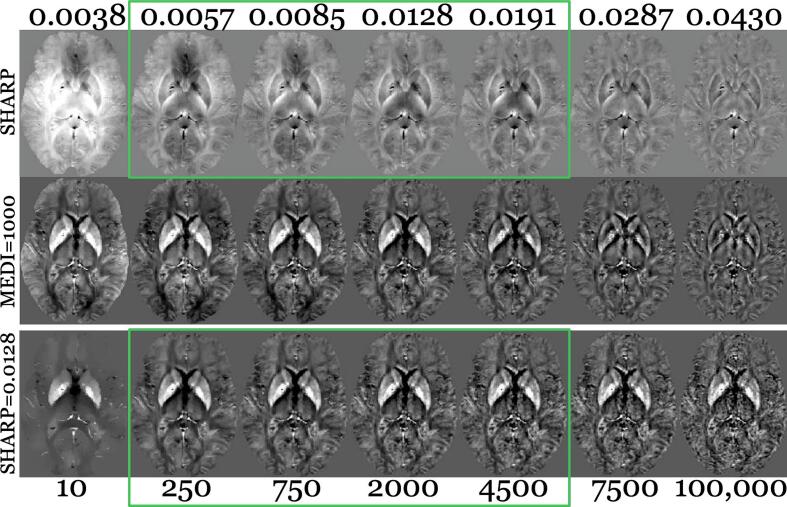
Field and susceptibility maps of a representative subject from the younger cohort (38 years old female; RMS subject [EDSS = 0]). Top row: Background corrected field maps over a range of alternative λ_B_ values listed above the top row. Middle row: Respective susceptibility maps (λ_D_ = 1000). Bottom row: Susceptibility maps over a range of alternative λ_D_ values listed below the bottom row, λ_B_ = 0.0128. Green boxes indicate acceptable λ_B_ and λ_D_ values, respectively. Contrast range: Field = −1 (black) to +1 rad (white); Susceptibility map = −0.08 to 0.15 ppm. (For interpretation of the references to colour in this figure legend, the reader is referred to the web version of this article.)

Fig. 1 shows the background-corrected field maps (top row) across varying λ_B_ values, their corresponding susceptibility maps with λ_D_ = 1000 (MEDI’s default; middle row), and susceptibility maps with different λ_D_ values at λ_B_ = 0.0128 (one of the visually acceptable phase images; bottom row). Under-regularization was observed at λ_B_ values between 0.0031 and 0.0038, while over-regularization occurred at λ_B_ values between 0.0287 and 0.0430. Similarly, λ_D_ values between 1 and 10 resulted in under-regularization, whereas values between 7,500 and 100,000 led to over-regularization. Acceptable λ_B_ (0.0057–0.0191) and λ_D_ (250–4500) values were identified (green boxes in Fig. 1) and used for subsequent analyses.

**Fig. 2:**
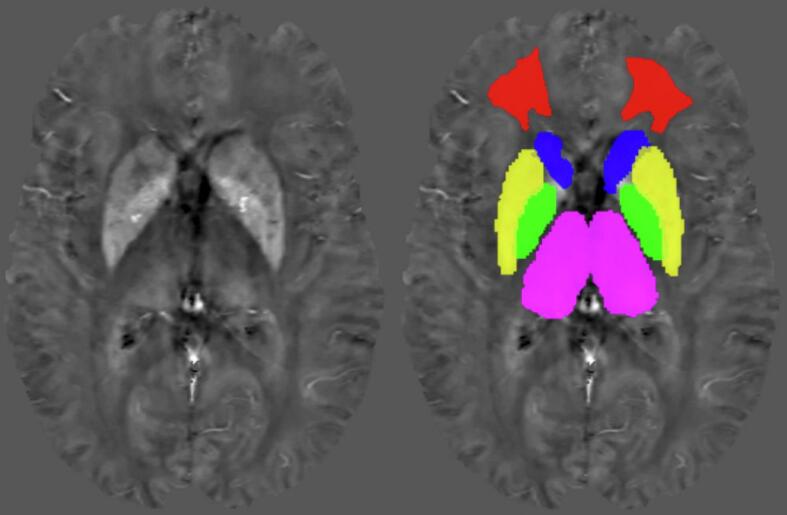
Representative susceptibility map from the younger cohort (41 years old female; RMS subject [EDSS = 1.5]; left) with DGM ROIs (violet = thalamus; green = GP; yellow = putamen; blue = caudate; right) and the FDWM ROI (red; referencing region) obtained from the study-specific template (Supplementary Fig. 2) in native space from the λ_B_/λ_D_ = 0.0128/1000 pipeline. Susceptibility map contrast range: −0.08 ppm (black) to 0.15 ppm (white). (For interpretation of the references to colour in this figure legend, the reader is referred to the web version of this article.)

Fig. 2 illustrates the DGM label segments from FIRST and the FDWM segment from the study-specific template on a representative susceptibility map, demonstrating accurate ROI placements (right side).

### 3.3 Findings in the younger cohort

**Fig. 3:**
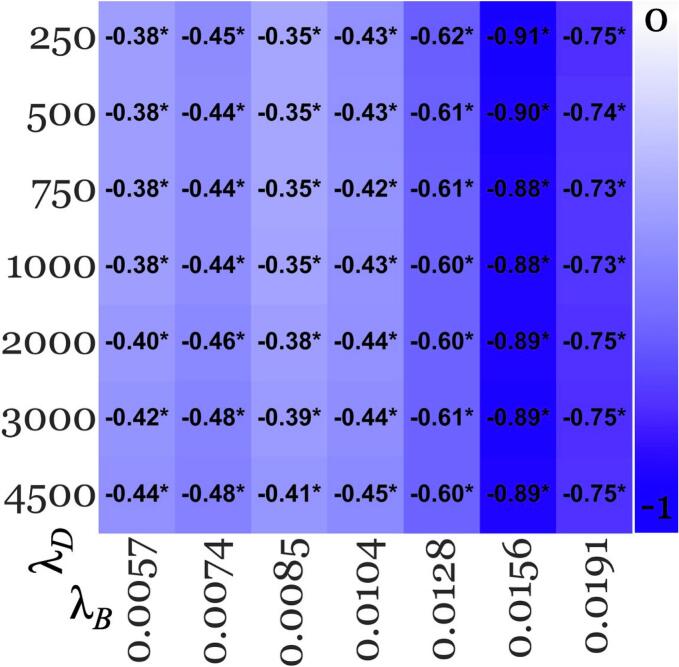
Age-corrected effect sizes for thalamus from acceptable pipelines (green boxes in Fig. 1) in the younger cohort. Lower values (−1; blue – falsifies the “early rise” hypothesis) represent higher absolute negative effect size, and red vice versa, with white for effect size of 0. Each row corresponds to a MEDI regularization parameter value (λ_D_, listed on the left-hand side), while each column represents a SHARP regularization parameter value (λ_B_, listed at the bottom). Significant findings were marked with asterisks (*). (For interpretation of the references to colour in this figure legend, the reader is referred to the web version of this article.)

**Fig. 4:**
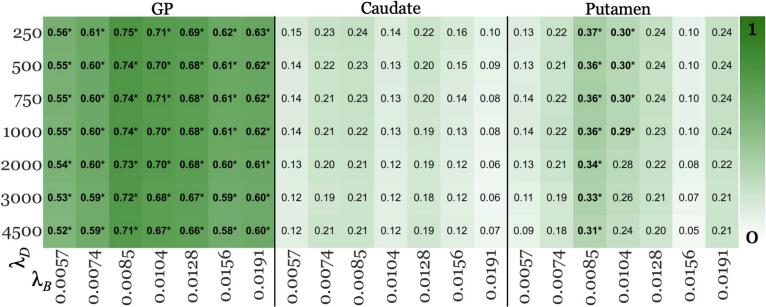
Age-corrected effect sizes for GP, caudate, and putamen, respectively, from acceptable pipelines (green boxes in Fig. 1) in the younger cohort. Higher values (+1; green) represent higher effect size, vice versa for white (0). Each of the three vertical panels summarizes results for one specific DGM region, listed at the top. For each panel, each row corresponds to a regularization (λ_D_) parameter used for MEDI (listed on the left-hand side), while each column represents a regularization (λ_B_) used for SHARP (listed at the bottom). Significant findings were marked with asterisks (*). (For interpretation of the references to colour in this figure legend, the reader is referred to the web version of this article.)

Fig. 3, Fig. 4 summarize the thalamic and other regional effect sizes across pipelines, respectively.

Decreased susceptibility in the thalamus of pwMS

Thalamic susceptibility was consistently reduced in pwMS compared to controls, with effect sizes ranging from −0.35 to −0.91. Effect sizes remained relatively stable across variations in MEDI’s λ_D_ but were substantially impacted by changes in SHARP’s λ_B_. The largest thalamic effect sizes were observed at the higher end of the acceptable λ_B_ range, with λ_B_ = 0.0156 yielding effect sizes between −0.88 and −0.91 across λ_D_ values. The pipeline that yielded the smallest absolute group difference was λ_B_/λ_D_ = 0.0191/250, with a difference of 3.9 ppb (controls: 7.7 ± 4.6 ppb, patients: 3.8 ± 5.5 ppb).

Increased susceptibility in other regions of pwMS

Across all pipelines, pwMS exhibited significantly higher susceptibility in the GP. In putamen, increased susceptibility in pwMS reached significance only for λ_B_ = 0.0085 and 0.0104 across the majority of λ_D_ values (Fig. 4; exception: λ_B_ = 0.0104 with λ_D_ = 2000, 3000, and 4500). No significant group differences were observed in the caudate across any pipeline.

### 3.4 Effect of excluding people with CIS from younger cohort

**Fig. 5:**
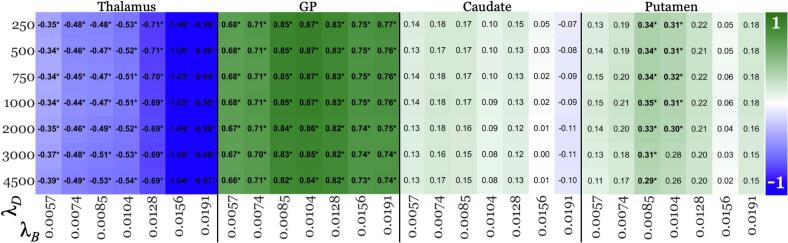
Age-corrected effect sizes for each region listed at the top, from acceptable pipelines (green boxes in Fig. 1) in the younger cohort with only RMS subjects. Lower values (−1; blue – falsifies our “early rise” hypothesis for the thalamus) represent higher absolute negative effect size, vice versa for green (+1), and white for effect size of 0. Each row corresponds to a specific regularization (λ_D_) parameter used for MEDI (listed on the left-hand side), while each column represents a regularization (λ_B_) used for SHARP (listed at the bottom). Significant findings were marked with asterisks (*). (For interpretation of the references to colour in this figure legend, the reader is referred to the web version of this article.)

Excluding individuals with CIS and restricting the analysis to RMS subjects did not change the overall study findings, as shown in Fig. 5.

### 3.5 Findings in the older cohort

Both pipelines, mentioned in Section 3.3 (under Decreased susceptibility in the thalamus of pwMS), showed significantly lower thalamic susceptibility in older pwMS compared to controls (*P* < 0.05), consistent with the original study’s findings (Table 4).

**Table 4:** Magnetic susceptibility (parts-per-billion [ppb]) for controls and pwMS from λ_B_/λ_D_ = 0.0156/1000 (highest thalamus effect size in the younger cohort; middle panel) and λ_B_/λ_D_ = 0.0191/250 (*smallest* absolute thalamus group difference in the younger cohort; bottom panel) in controls and pwMS of this study, compared to the original study findings by Pudlac et al. (top column, left) and Rudko et al. (top column, right), respectively. Rudko et al. originally reported regional standard errors (SE) which were converted to standard deviations (SD) using their original sample size (*N*; SD=SE*sqrt(*N*)). Note: All regional values are mean ± SD; Significant findings (*P ≤* 0.05) are bold-faced with asterisks (*).

Susceptibility (ppb)	Pudlac et al.			Rudko et al.		
Regions	Controls	pwMS	*P*	Controls	pwMS	*P*
Thalamus	16.1 ± 8.2	10.8 ± 5.6	0.002*	25.9 ± 15.6	37.3 ± 10.5	<0.001*
GP	83.7 ± 11.1	83.6 ± 16.1	0.21	128.5 ± 10.6	141.5 ± 21.5	<0.001*
Putamen	30.7 ± 7.4	30.3 ± 11.7	0.34	33.7 ± 3.9	42.1 ± 10.4	0.008*
Caudate	41.1 ± 10.1	39.3 ± 10.8	0.59	39.7 ± 9.1	45.4 ± 7.2	0.003*
Present Study						
λ_B_ = 0.0156 & λ_D_ = 1000						
Thalamus	9.6 ± 10.7	4.0 ± 9.5	0.03*	5.4 ± 6.1	-1.1 ± 7.8	<0.001*
GP	143.1 ± 23.9	154.4 ± 23.9	0.05*	82.7 ± 17.8	94.5 ± 20.3	<0.001*
Putamen	63.2 ± 18.5	59.2 ± 18.8	0.22	34.1 ± 9.0	35.0 ± 10.1	0.30
Caudate	63.2 ± 14.6	64.8 ± 11.8	0.34	40.2 ± 7.6	41.1 ± 10.5	0.21
λ_B_ = 0.0191 & λ_D_ = 250						
Thalamus	14.0 ± 8.1	10.0 ± 7.2	0.03*	7.7 ± 4.6	3.8 ± 5.5	<0.001*
GP	115.9 ± 21.2	126.0 ± 21.1	0.05*	62.6 ± 15.4	73.2 ± 17.7	<0.001*
Putamen	42.9 ± 16.4	39.9 ± 16.8	0.25	19.0 ± 7.2	20.8 ± 8.0	0.10
Caudate	47.7 ± 13.6	47.1 ± 9.6	0.43	30.9 ± 7.1	31.6 ± 8.9	0.27

In the GP, both pipelines showed significantly higher susceptibility in pwMS compared to controls (+11.3 ppb and + 10.1 ppb, respectively; *P* ≤ 0.05). In contrast, no signficiant group differences were observed in the putamen or caudate.

## 4 Discussion

Our study aimed to determine whether younger pwMS exhibit increased average thalamic susceptibility. This outcome would support the previously formulated “early-rise late-decline” hypothesis (Schweser et al., 2018). We did not find increased thalamic susceptibility in pwMS. Instead, both the younger and the older cohorts demonstrated reduced thalamic susceptibility compared to controls. However, we observed increased magnetic susceptibility in all other DGM regions, consistent with Rudko et al.’s findings. We also observed that all regional outcomes were consistent across various QSM algorithmic configurations (λ_B_ and λ_D_ values), though they presented with variable effect sizes (Fig. 3, Fig. 4, Fig. 5). Furthermore, findings remained consistent after replacing CIS subjects with RMS subjects.

Our study suggests that differences in QSM processing parameters are unlikely to explain the discrepancy between our results and those of Rudko et al. This conclusion aligns with the original study’s corroboration of QSM findings using R_2_* mapping. Despite closely matching cohorts based on reported clinical and demographic characteristics, residual differences between study populations represent the most likely explanation for the discrepancy. To the author’s best knowledge, all studies reporting increased thalamic iron levels, including that of Rudko et al., were conducted by Canadian research groups (Rudko et al., 2014, Cobzas et al., 2015); and none have reported decreased thalamic susceptibility (Elkady et al., 2017, Elkady et al., 2019, Fujiwara et al., 2017). Thus, discrepancies may stem from geographic factors, such as environmental, cultural, or lifestyle factors—known to affect iron metabolism (Gustavsson et al., 2024, Ahern et al., 2024, Zachariou et al., 2025)—or differences in clinical treatment protocols. Additionally, variations in the control cohort characteristics, rather than pwMS cohorts, may have influenced study outcomes. For instance, recruitment strategies may have selected controls from different socioeconomic groups or ethnicities compared to pwMS groups. These factors are often not recorded or reported in the literature, limiting the reproducibility of studies.

Furthermore, study differences may not reflect iron homeostasis but could instead stem from greater thalamic atrophy in pwMS in the original study than in ours. Recent studies indicate that gray matter atrophy can alter susceptibility measurements independently of cellular iron homeostasis (Schweser et al., 2021, Hernández-Torres et al., 2019).

Secondary outcomes

This replication study provides a thorough assessment of how QSM processing factors influence clinical study outcomes, involving the computation of close to 30,000 susceptibility maps. Contrary to the belief that QSM-based study outcomes dependent heavily on regularization parameters settings (Salman et al., 2024); we found that key findings (statistically significant group differences; Fig. 3, Fig. 4, Fig. 5) remained consistent across all acceptable SHARP and MEDI parameter choices. Overall, this observation enhances the confidence in the comparability and reproducibility of clinical research using QSM. Nevertheless, our results highlight the importance of optimizing BFR regularization parameters to maximize statistical power for detecting group differences. The effect sizes in our study were mainly affected by the SHARP parameter, with up to 160 % variation between λ_B_ values (Fig. 3). In contrast, the impact of the MEDI regularization parameter, λ_D_, was much lower (≤15 %; Fig. 3). SHARP regularization strongly affects the degree to which slowly varying inhomogeneities, such as residual background fields, are maintained in the corrected field map (Özbay et al., 2017, Salman et al., 2025). These slowly varying inhomogeneities, which are not always discernible during quality control (Salman et al., 2025); can propagate through the dipole inversion into the susceptibility map, where they may have a substantial effect on regional average susceptibility values, as shown previously (Özbay et al., 2017, Salman et al., 2025). Conversely, dipole inversion regularization, which primarily reduces noise and streaking artifacts, appears to have minimal impact on regional susceptibility values, at least for MEDI. The increased sensitivity of the effect sizes to BFR regularization compared to dipole inversion regularization indicates that placing additional emphasis on optimizing the regularization parameter of the BFR algorithm may enhance the reliability of QSM studies (Langkammer et al., 2018, QSM Challenge 2.0 Organization Committee et al., 2021).

## 5 Limitations

Differences in thalamic subnucleus atrophy between study cohorts may also contribute to discrepancies (Trufanov et al., 2021, Magon et al., 2014, Blyau et al., 2023, Krijnen et al., 2024, Koubiyr et al., 2024, Levy et al., 2022). Investigating of these effects could provide a more nuanced understanding of intra-thalamic iron heterogeneity in pwMS (Schweser et al., 2018, Elkady et al., 2018, Cagol et al., 2024). However, investigating thalamic sub-nuclei was beyond the scope of this study.

Differences in field strength, equipment, and pulse sequence parameters, such as echo times, flip angle, and repetition time, may also contribute to the discrepancy between our findings and those of Rudko et al. Specifically, it is possible that microstructural and multicompartment effects, which are unaccounted for in current QSM algorithms (Wharton and Bowtell, 2015, Schweser and Zivadinov, 2018) and neglected in monoexponential R_2_* mapping (Du et al., 2007, Sati et al., 2013); manifest differently in the reconstructed maps. Depending on field strength and pulse sequence parameter differences, these effects may result in a systematic bias manifesting in the observed differences between study outcomes. In particular, the thalamus is a region with relatively high myelination and complex substructure (Deistung et al., 2013); which makes it especially susceptible to microstructural and multicompartment effects. Ultra-high-field studies at 7T have expanded possibilities for detailed thalamic sub-nuclei imaging (Deistung et al., 2013, Alkemade et al., 2020, Martinez et al., 2024); primarily due to the enhanced signal-to-noise ratio (SNR) and increased sensitivity to susceptibility contrast. However, evidence suggests that QSM outcomes are relatively robust across various scanner models, pulse sequences, and field strengths, with several studies indicating consistent reproducibility (Rua et al., 2020, Spincemaille et al., 2020, Spincemaille et al., 2019, Lancione et al., 2022). Nonetheless, further investigation is necessary to comprehensively understand how these technical factors—especially field strength and pulse sequence configurations—may influence group differences and overall susceptibility quantification.

## 6 Conclusion

Our study did not replicate previous reports of increased thalamic susceptibility in young people with MS. Instead, our findings indicate that declining thalamic susceptibility may occur early in the disease course. These findings contradict the “early-rise late-decline” hypothesis of thalamic susceptibility in MS. Further investigation is required to determine the factors influencing inter-subject variability in thalamic susceptibility and to evaluate its potential as an early biomarker for MS.

## CRediT authorship contribution statement

**Fahad Salman:** Writing – review & editing, Writing – original draft, Visualization, Validation, Software, Project administration, Methodology, Investigation, Formal analysis, Data curation, Conceptualization. **Niels Bergsland:** Writing – review & editing, Resources, Data curation. **Michael G. Dwyer:** Writing – review & editing, Resources, Data curation. **Jack A Reeves:** Data curation. **Abhisri Ramesh:** Software. **Dejan Jakimovski:** Data curation. **Bianca Weinstock-Guttman:** Data curation. **Robert Zivadinov:** Writing – review & editing, Resources, Data curation. **Ferdinand Schweser:** Writing – review & editing, Writing – original draft, Validation, Supervision, Software, Resources, Project administration, Methodology, Investigation, Funding acquisition, Conceptualization.

## Funding

Research reported in this publication was performed in part at the University at Buffalo’s Center for Computational Research (Center for Computational Research) (NIH award S10OD024973 and NSF award 1724891), and was supported by the National Institute of Neurological Disorders and Stroke of the National Institutes of Health under Award Number R01NS114227 and the National Center for Advancing Translational Sciences of the National Institutes of Health under Award Number UL1TR001412. The content is solely the responsibility of the authors and does not necessarily represent the official views of the National Institutes of Health.

## Declaration of competing interest

The authors declare the following financial interests/personal relationships which may be considered as potential competing interests: Robert Zivadinov has received personal compensation from Bristol Myers Squibb, EMD Serono, Sanofi, Mapi Pharma, Sana Biotechnologies and Filterlex for speaking and consultant fees. He received financial support for research activities from Bristol Myers Squibb, EMD Serono, Mapi Pharma and Protembis and Filterlex. Michael G. Dwyer received personal compensation from Bristol Myers Squibb, Novartis, EMD Serono and Keystone Heart, and financial support for research activities from Bristol Myers Squibb, Novartis, Mapi Pharma, Keystone Heart, Protembis, and V-WAVE Medical.

## Data Availability

Computer code used for the processing of QSM data in this manuscript has been made available at https://doi.org/10.5281/zenodo.13375700. The imaging data supporting the findings of this study are available from the corresponding author upon reasonable request and subject to institutional review board approval. These data are not publicly accessible due to privacy and ethical considerations.
