# Supplementary material for: Thalamic iron in multiple sclerosis: Waning support for the early-rise late-decline hypothesis
Source: Neuroimage Clin. 2025 Mar 26;46:103771. doi: 10.1016/j.nicl.2025.103771 (PMC12002950; doi:10.1016/j.nicl.2025.103771)
Supplement: Supplementary Data 1 [file mmc1.docx]

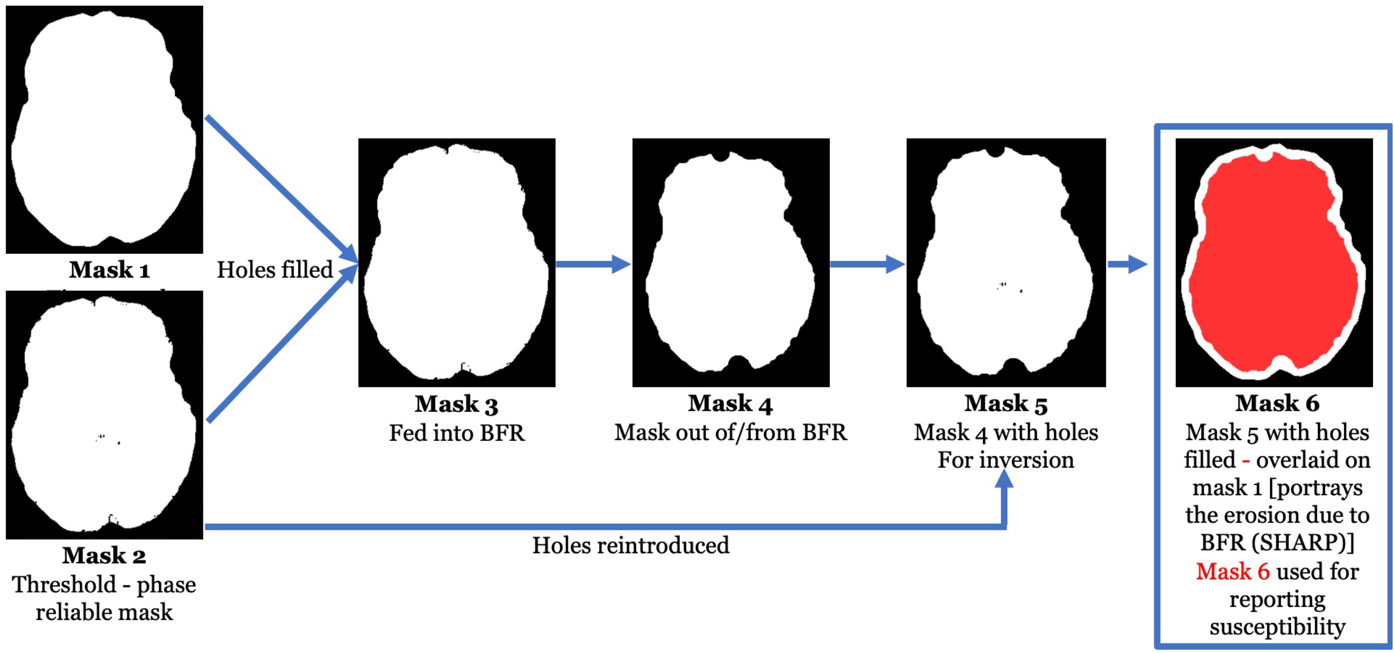


Supplementary Figure 1. Schematic of the masking procedure utilized to generate susceptibility maps. Displays a mask obtained from a representative healthy subject within the study cohort.


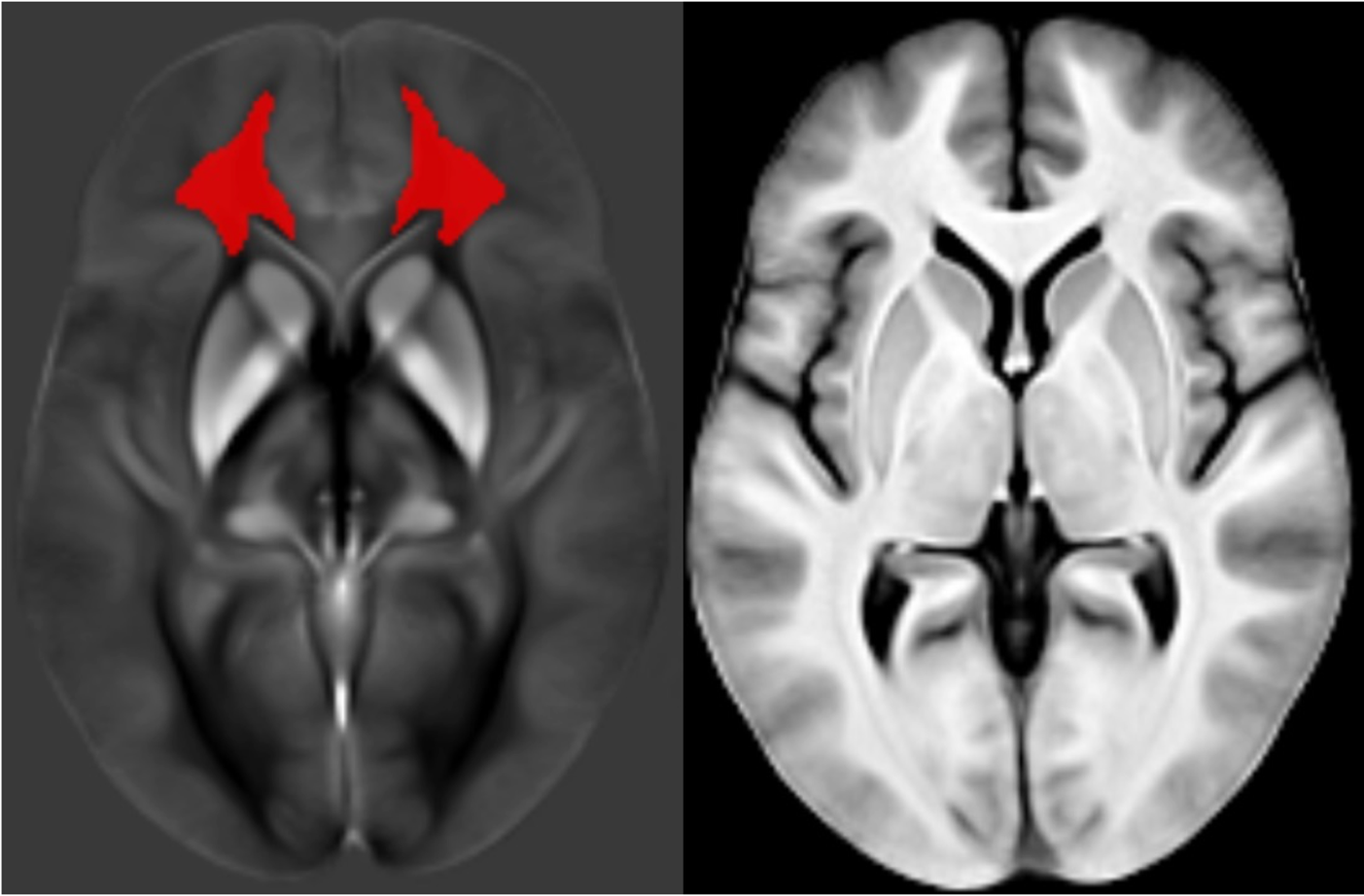


Supplementary Figure 2. Susceptibility and T1w contrasts of the study-specific template generated using the bi-parametric approach, respectively. Manually delineated FDWM (red) label is shown on the susceptibility contrast of the template. The contrast for the susceptibility template was set to -0.04 (black) to 0.14 (white) ppm.
